# Traditional management of microorganisms in fermented beverages from cactus fruits in Mexico: an ethnobiological approach

**DOI:** 10.1186/s13002-019-0351-y

**Published:** 2020-01-10

**Authors:** César I. Ojeda-Linares, Mariana Vallejo, Patricia Lappe-Oliveras, Alejandro Casas

**Affiliations:** 1 Instituto de Investigaciones en Ecosistemas y Sustentabilidad, Universidad Nacional Autónoma de México, Campus Morelia. Antigua Carretera a Pátzcuaro 8701, Col. San José de la Huerta, Morelia, Michoacán 58190 México; 2 Instituto de Biología, Universidad Nacional Autónoma de México, Circuito Exterior, Ciudad Universitaria, Ciudad de México, C.P. 04510 México

**Keywords:** Columnar cacti, Ethnozymology, Fermentation practices, Foodways, Colonche, *Opuntia*

## Abstract

**Background:**

Fermentation is an ancient technique for preserving and improving the qualities of food and beverages throughout the world. Microbial communities, not seen by the producers of fermented goods, are the actors involved in the fermentation process and are selected upon through different management processes in order to achieve a final product with culturally accepted features. This study documented the preparation of “colonche” which is a type of traditionally fermented beverages made with the fruits from several cactus species in two main producing regions of Mexico, the Altiplano and the Tehuacán Valley. We documented the selection processes of the cactus species used and the practices that could influence microbial community composition, as well as, how the producers reach the desirable sensorial attributes of the beverages.

**Methods:**

We conducted 53 semi-structured interviews and participatory observations with colonche producers in 7 communities of the Altiplano and the Tehuacán Valley in order to characterize the practices and processes involved in the elaboration of the beverage. *Opuntia* and columnar cacti species used in colonche production were collected during fieldwork and identified. Selected sensorial attributes of *Opuntia* colonches were characterized by a ranking table and visualized by principal component analysis in order to distinguish differences of this beverage in the Altiplano localities.

**Results:**

Thirteen cactus species are used for colonche production in both regions studied. In the Altiplano, the most commonly used fruit is *Opuntia streptacantha* because it contributes to the preferred attributes of the beverage in this region. Selection of substrates by producers depends on their preference and the availability of fruits of *O. streptacantha* and other species. Fermentation is mainly conducted in clay pots which is perceived to be the best type of vessel contributing to the preferred sensorial properties of colonche. The two main differences in colonche preparation between the villages are the practice of boiling the fruit juice and the use of pulque (fermented sap of *Agave* species) as inoculum. The most contrasting sensorial attributes selected between localities are the alcohol content and sweetness, which might be in accordance with the practices used for obtaining the final product. Colonche is produced mainly for direct consumption and secondarily used as a commercialized good to be sold for economic gains contributing to the general subsistence of households. The preparation methods are passed on by close relatives, mainly women.

**Conclusions:**

Traditional producers of colonche use several techniques in order to reach specific sensorial attributes of the final product. The production of colonche has been upheld for generations but fermentation practices are divided into two categories; (1) the use of an inoculum (either from pulque, or from colonche saved from the previous year), and (2) the use of “spontaneous” fermentation. The differing practices documented reflect the contrasts in the preferred sensorial attributes between regions. Colonche is a beverage that contributes to regional pride, cultural identity and is appreciated because of its gastronomic value. Here, we argue that there is a clear relationship of human knowledge in the management of microbiota composition in order to produce this beverage. In-depth documentation of the microbiota composition and dynamics in colonche will contribute to the preservation of this valuable biocultural heritage.

## Introduction

It has been estimated that nearly one-third of food and beverages in human diet involves fermentation processes [1]. Some fermented products are widely distributed and support dynamic industries over a growing market for functional foods [2]. However, most of them are locally distributed in rural areas and are rarely seen or absent in urban contexts and markets. The ancient process of fermentation has been utilized for a broad spectrum of substrates dating back to approximately 9000 years ago from pottery [3], but their use and management could have occurred even earlier.

Fermentation is an outstanding strategy enabling people to preserve foods as well as to transform edible raw matter into new products with unique sensorial properties [4, 5]. It also enhances the nutritional value of food and beverages that are prepared through fermentation processes representing an important part of human nutrition in practically every food culture of the world [4]. Although the microbial relationship with food production, knowledge, health, and heritage, is highly important, relatively few studies have been directed to characterize the microbial landscape that is composed of aggregated culinary and agricultural management decisions.

An understated aspect of ethnobiological studies is the knowledge and use of microbiota for traditional fermentation practices involving the autochthonous microbiota found on plant ingredients and from other natural sources. Nevertheless, the transformation of raw materials into value-added products could be assessed in a variety of ecological niches. Relatively scarce attention has been centered on plant attributes and management over fermentation practices to produce final products. Nowadays, the loss of this specialized knowledge is relevant because it is associated with the loss of biodiversity and practices associated with communities that use fermented foods to bolster food security, foster culturally important tastes, or anchor connections between food, identity, and health [6–8].

Given the implications of this lack of information, it is vital to extend empirical studies to different systems. Information on spontaneous traditional fermented beverages prepared and consumed in Mexico is scarce, mainly dominated by ferments of agave and maize. For example, in Mexico, “colonche” (also referred to as *coloche* and *nochoctli*) is a group of fermented beverages produced by fruits of different cactus species, which has yet to be documented in detail in published literature. Colonche is a term resulting from the Spanish deformation of the indigenous Nahuatl term *nochoctli* which is derived from the words *nochtli* used for naming cacti fruits, and *octli* the term used for naming the fermented sap of agaves.

Fresh or fermented cactus fruits were widely consumed by indigenous peoples [9–12]. In particular, colonche was produced and consumed in pre-Columbian times by the indigenous peoples called “Chichimeca” by the Aztecs from the northern region of Mexico [13, 14]. In villages of the Tehuacán Valley, colonche is prepared with fruits of *Opuntia* and several columnar cacti species like *Pachycereus weberi*, *Escontria chiotilla*, *Stenocereus* spp., and *Polaskia* spp. [15–18]. Additionally, other types of fermented cacti fruit juices have been recorded in other regions and were consumed by the Aztecs and their tributary peoples from northern to southern Mexico [15–18]. In the Sonoran Desert and northwestern California, fruits, seeds, and ferments from saguaro, *Carnegiea gigantea*, and cardón, *Pachycereus pringlei*, were consumed by the Papago indigenous people [19, 20]. Fruits from these cacti were crushed and squeezed for juice, then, the fruit juice was boiled and ultimately fermented resulting in the product named “sahuaro” [21, 22]. Although these wines do not receive the name of colonche, since the regional human cultures were not influenced by the Aztec, this beverage is clearly similar, and names are mainly generic. Nevertheless, scarce information is available on these beverages and the production of this traditional fermented beverage is decaying [23].

Plants of the genus *Opuntia* are the most abundant group of the Cactaceae family, currently spreading throughout the Americas, Europe, Asia, Africa, and Australia [24–26]. The cactus prickly pear fruit is oval elongated berries, with thick pericarp, juicy pulp with numerous seeds, and a semi-hard rind with thorns. The pericarp and edible pulp may have different colors such as green, greenish-white, canary yellow, lemon yellow, orange, red, cherry-red, or purple hues [27–29]. Prickly pears have long been known in traditional medicine for treating several pathologies such as ulcers, dyspnea, and glaucoma, as well as liver illnesses, wounds, and fatigue [30, 31]. Recent studies have found that juice from red prickly pear fruits has anticlastogenic potential because of their high number of antioxidants [32]. In addition, columnar cacti have been recorded as an important source of water, food, vegetables, and medicine [33].

In order to address the knowledge and use of microbiota in traditional fermentation practices, we examined the variation of processes involved in colonche production in two regions of Mexico. The selection of fruits, fermentation practices, and sensorial preferences was registered. We selected the traditional fermented beverage known as colonche because it is distributed throughout different regions, it is poorly studied and has been registered as a beverage at risk of extinction [34]. Colonche will allow us to compare the importance of traditional knowledge of fermentation in several localities because it is thought to vary depending on local cultural preferences. Our study looks to test the prediction that traditional ecological knowledge of fermentation practices is still in effect today but in decay. Additionally, our aim is to show that final fermented products are shaped by the selection of substrates, practices, tools and that the quality of these products is culturally shaped and based on sensorial attributes.

## Materials and methods

### Study area

**Fig. 1.**
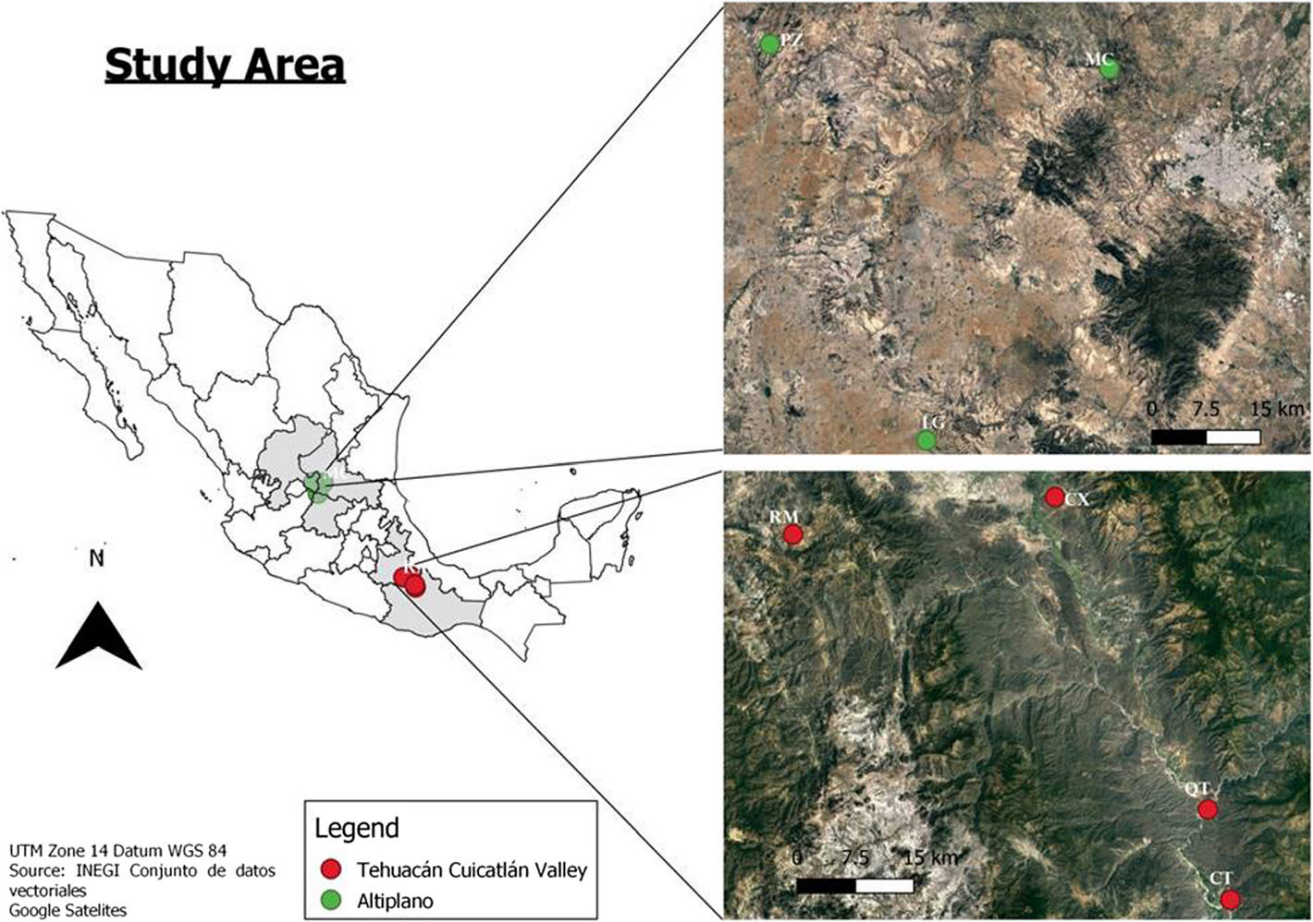
Study area in Mexico, grey regions comprise states where colonche production has been documented. Red points are sites where information was collected in the Tehuacán Valley; green points belong to the Altiplano localities. The map was elaborated with an open-source software, Qgis3.4, https://qgis.org/es/site/. Source: INEGI conjunto de datos vectoriales

Local stakeholders were identified in three localities of the Altiplano region and four communities of the Tehuacán-Cuicatlán Valley region. The snowball method was followed in order to identify colonche producers in each locality. Producers who gave their permission were then interviewed. In the Altiplano region, we studied the communities of Laguna de Guadalupe (LG), in the state of Guanajuato, Mexquitic de Carmona (MC) in the state of San Luis Potosí, and Pinos (PZ) in the state of Zacatecas. In the Tehuacán-Cuicatlán region, we studied the communities of Los Reyes Metzonla (RM) and Coxcatlán, in the state of Puebla, and Quiotepec and Cuicatlán in the state of Oaxaca (Fig. 1). The climate in both regions is semiarid and characterized by a cold winter and a hot summer. The vegetation is predominantly xerophytic and dominated by arborescent cacti (mainly *Opuntia* spp. and columnar cacti) [35]. The Altiplano region was historically called the “Tunal grande,” a term that refers to the high availability of cactus prickly pear fruits, whereas the vegetation of the Tehuacán Valley is predominantly columnar cacti forests [36].

### Ethnobiological study

We performed semi-structured interviews and participatory observations in order to document the preparation process of colonche, the different plant species substrates, sources of inoculation, and control of microbiota responsible for fermentation. We also documented information about the economic role of colonche production in households, the purpose of preparing this beverage, the most important attributes valued in their final product, and socio-cultural data related to the consumption of colonche. The semi-structured interviews were guided through a questionnaire (Additional file 1), including the following topics: (1) general information about producers, (2) the production process, emphasizing practices for fermentation (3) tools, instruments and techniques employed, (4) species involved providing substrates, (5) availability of plant substrates (fruits available in the neighboring territories), and (6) perception about sensorial attributes of colonche. In relation to the latter topic, information was obtained by inquiring directly to rank the importance of traits.

### Sampling

Fifty-three colonche producers were interviewed. Thirty-one of the producers were located in three localities of the Altiplano during the production season (August-September), 71% of the producers were elder people (40 to 70 years old). The remaining 22 producers were located in four communities of the Tehuacán-Cuicatlán region (in April and May) (Table 1). Finally, a principal component analysis (PCA) was performed to characterize the most valuable attributes in the final product for each locality; this analysis was performed through the Rstudio software.

**Table 1.** Number of producers interviewed in both regions

Region	Community	Men	Age	Women	Age
Altiplano	Laguna de Guadalupe (LG)	7	> 40	1	> 40
		2	< 40	4	< 40
	Mexquitic de Carmona (MC)	3	> 40	6	> 40
		2	< 40	3	< 40
	Pinos (PZ)	2	> 40	1	> 40
Tehuacán Valley	Los Reyes Metzontla (RM)	3	> 40	2	> 40
	Coxcatlán (CX)	4	> 40	1	> 40
	Quiotepec (QT)	4	> 40	3	> 40
	Cuicatlán (CT)	3	> 40	3	> 40

### Botanical collections

*Opuntia* and columnar cacti species used for colonche production were collected and identified by local members of the communities studied. Then, *Opuntia* species of the Altiplano region were identified with the help of Dr. Antonio Reyes-Agüero from the Autonomous University of San Luis Potosí, an expert in *Opuntia* taxonomy. The columnar cacti species were identified by our research team.

## Results

### General information of colonche producers

Colonche production in LG is mainly produced by men, while in MC production is predominantly carried out by women (Table 1). In order to avoid noise in the data, the information from the PZ was removed from our study because of the low sample size and unclear production methods.

Colonche producers in the Altiplano region stated that the beverage is mainly consumed by family members and secondarily sold representing a small source of income (less than 10% of their total income during *Opuntia* fructification. Colonche has such high value that consumers have found ways to store and transport the beverage to the USA and other regions of Mexico, with the main purpose to share with members of their families.

It is important to highlight that most of the producers learn the production process from their mothers. Also, new producers argue that it was a beverage that their family ceased to produce due to the absence of the mother. Nowadays, they have, once again, began to produce colonche because of its symbolic association with family relations. However, as a main threat for continuing colonche production, the producers interviewed identified the disinterest in colonche production and consumption by the young people, mainly because “it represents more work” and it is not easily available as other beverages like beer. Above all, producers preferred colonche consumption and recognize that the attitude upheld by the younger generations and the substitution of colonche for other beverages like beer endanger the continuation of the production of this historic product.

Colonche is not considered as a medicinal beverage in most of the communities, only 7% of the respondents considered that it has health benefits related to lungs and stomach illnesses, while most of the respondents said that they were not aware of health-related benefits. Most of the respondents consider that colonche has euphoric attributes due to its alcohol content.

In MC, colonche consumption occurs mostly during Sunday’s market; therefore, the production of colonche begins Wednesday. Each household produces at least 20 L to bring to the Sunday market. Unlike MC, colonche in LG is locally consumed within their households and shared with friends and guests. However, a recent festival named “feria del colonche,” which takes place in LG during September has proven to be a very successful way to popularize and share colonche in the region and generate incomes for local producers.

In all villages of the Tehuacán-Cuicatlán Valley, colonche is produced for direct consumption by household members, family meetings and parties. Additionally, colonche is a sub-product associated with the extraction of the seeds of *P. weberi*, which are highly valued in regional markets [37, 38].

### Substrate selection

A total of seven *Opuntia* species and varieties and six columnar cacti species are used for colonche production in both regions. However, as mentioned above, *Opuntia streptacantha* is the main ingredient of colonche produced in the Altiplano region and *Pachycereus weberi* in the Tehuacán Valley. Nevertheless, all the communities add fruits of other *Opuntia* species when fruits of *O. streptacantha* are not available. The other species of cactus prickly pears used for colonche production include *Opuntia orbiculata*, *O. robusta*, *O. hyptiacantantha*, *O. phaeacantha*, and *O. ficus-indica*. In particular, in LG, *O. streptacantha* is widely distributed in managed and unmanaged landscapes and producers invest at least 2 h (± 30 min) daily for fruit collection. In contrast, in MC, *O. streptacantha* is not widely distributed and is only found in private managed properties; as a result, producers propagate *O. streptacantha* by using cuttings, young plants, and cladodes to establish plants near their houses or within their gardens. If producers of colonche do not directly collect *O. streptacantha* fruits they purchase or exchange colonche with local growers for access to fruits. In contrast, fruits are widely available in PZ but, nowadays, is rarely produced. In fact, production in PZ was not well characterized because of the lack of producers and information in this region.

In the Altiplano localities, *Opuntia* prickly pear fruits are collected with a sickle and peeled with a knife in situ. Then, the peeled fruits are placed in plastic containers and transported to the house of the colonche producer. Harvesting can include all family members, and it is performed in the morning to avoid collecting fruits warmed by the sun. Ripe *Opuntia* fruits are selected for colonche production. Non-ripe fruits have less sugar content and are typically not preferred. If non-ripe fruits are used, more fruits must be collected in order to produce colonche with the same flavor quality. Additionally, red fruits are favored for colonche production, however, when red fruits are scarce, yellow and white fruits could be added resulting in changes in flavor, color, and texture (Fig. 3a).

In the Tehuacán Valley, people from Quiotepec and Coxcatlán prepare colonche (also called “pulque rojo” or “red pulque”) mainly with fruits of *Pachycereus weberi* or cardón, *Polaskia chichipe*, *Escontria chiotilla*, *Stenocereus stellatus*, *S. pruinosus*, and *Opuntia pilifera*. Fruits of these species are produced from late January to early May, the peak of fruit production occurs in April. The exceptions are *Escontria chiotilla* and *Stenocereus stellatus* which have fruit production peaks from July to September. In Cuicatlán (Cu), people use the fruit of *Opuntia pilifera* for preparing colonche. In this town, as well as in Quiotepec (Qu), and Coxcatlán (Co) people commonly use fruits of *E. chiotilla*. They say they occasionally prepare colonche with fruit of *S. pruinosus* and *S. stellatus*, but they say these fruits should be ripe, otherwise, the colonche would be bitter and sour. People said they also have tried colonche preparation with the fruit of *Hylocereus undatus*, and *Lemairocereus hollianus* but they do not like neither the insipid flavor nor the viscous texture of colonche resulting from the fruit of these species. In San Luis Atolotitlán (SLA) and Metzontla (Mz), people more frequently use fruit from *E. chiotilla*, *P. chichipe*, *P. chende*, *S. stellatus*, and *S. pruinosus*. In all cases, seeds are removed, but only recovered for consumption and commercialization the seeds of *S. pruinosus* and *S. stellatus*, particularly those varieties producing larger seeds. People gather cardón fruits by using a “chicol,” which is a long stick, commonly a “carrizo” stem (*Arundo donax*), whose extreme is cut into strips which are doubled and tied with a wire or palm fibers to form a basket, which is appropriate to pull the fruit down the cardón branches without causing damage to cactus plants.

All of the producers at the Altiplano quantify the yield of two plastic buckets of 25 L of peeled cactus prickly pear fruits that allow obtaining 15 ± 2 L of colonche. Whereas in the Tehuacán Valley, yields are similar, a 20-L bucket of peeled fruit may yield 16 to 17 L of juice, most probably because seeds of columnar cacti are smaller than those of *Opuntia* fruits. After being collected, fruits are carried from field to homes in buckets, where these are peeled outdoors and then pressed using a cloth, in order to separate juice and seeds, thus catching the seeds.

### Production process

**Fig 2.**
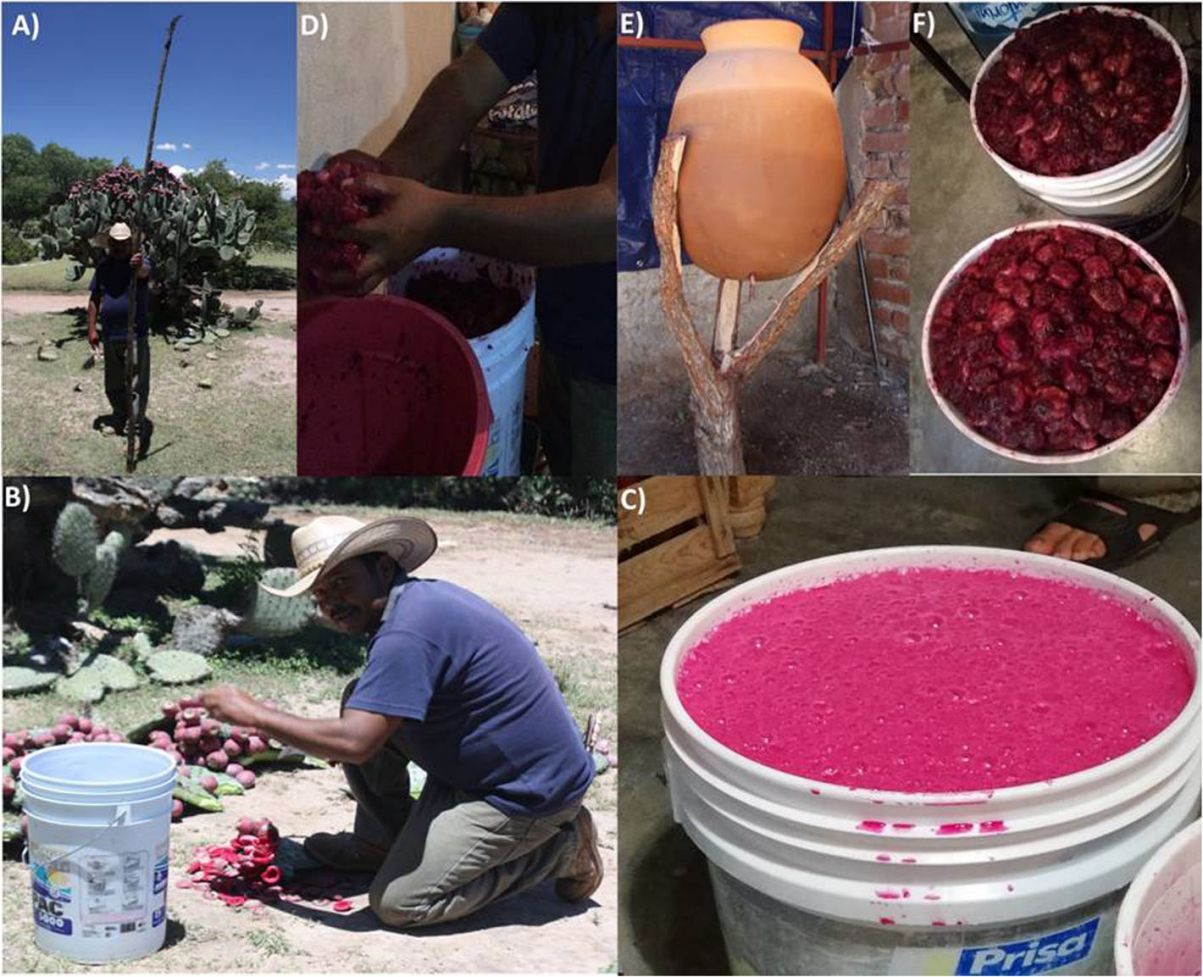
Colonche production at Laguna de Guadalupe (LG), Guanajuato. **a** Fruit harvesting. **b** Peeling the fruits in situ and transportation. **c** Peeled fruit storage. **d** Crushing and placing the fruits inside the clay pots. **e** 12 h fermentation inside the clay pot. **f** Colonche ready for consumption

Figure 4 illustrates the general process of colonche production and differences in both regions and localities. As mentioned above, in the Altiplano region *Opuntia* prickly pear fruits are collected and peeled in situ in the morning and transported to the houses by the colonche producers (Fig. 2a), then the peeled fruits are left outdoors exposed in plastic buckets (Fig. 2b). Undoubtedly, this exposure would facilitate the colonization of microorganisms for the fermentation process (Fig. 2c). Peeled fruits, slightly crushed by hand, are placed inside the clay pots in the evening every day (around 18:00 to 20:00 h), then the fruits are left to ferment in clay pot (Fig. 2d). As a safety practice, hands must be dried and cleaned without soap as the water and soap contaminate the colonche. The fermentation of colonche will be described in more detailed in the following section.

During the fermentation, clay pots are placed outdoors and covered with a thin cloth (Fig. 2e). Sometimes, clay pots are placed inside their homes during the night in order to maintain a constant temperature. If raining, clay pots are covered and moved to dry places. After the fermentation is completed, the seeds are removed with a sieve and colonche is freshly served. Colonche is stored in plastic buckets inside producer households and locally distributed (Fig. 2f). Part of the colonche batch that is not consumed fresh can be stored in plastic bottles and may include a mixture of several items as 2 L of colonche for 1 L of alcohol, pineapple, cinnamon, raisins, and anise. This mixture can be saved for multiple years or sold as “vino de tuna” or cactus prickly pear wine. Cactus prickly pear wine is a way to enjoy colonche during the part of the year when fresh colonche is no longer available. But, it should be mentioned that the process to make the “vino de tuna” significantly changes many sensorial attributes in comparison to fresh colonche.

**Fig. 3:**
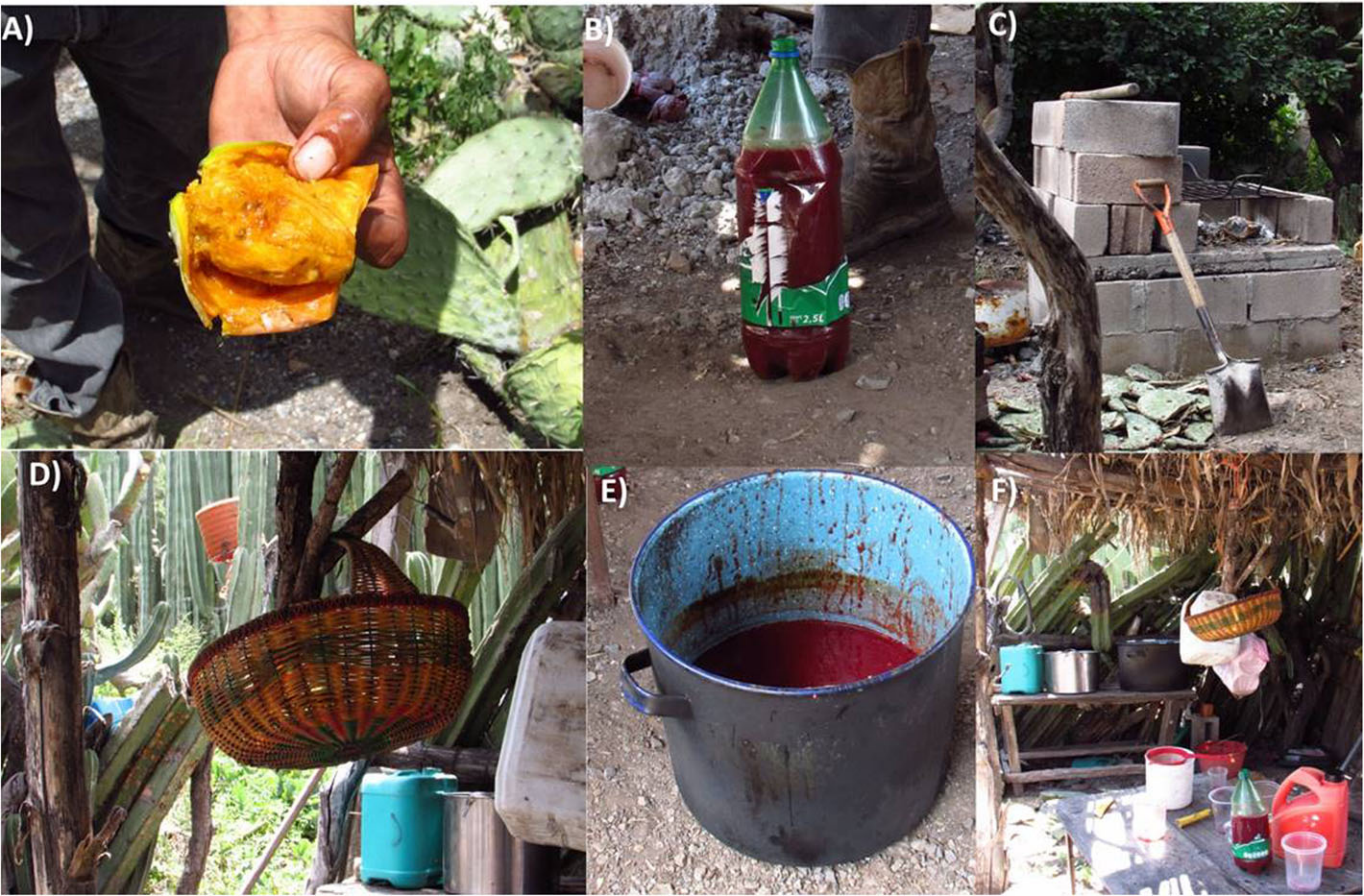
Colonche production in Mexquitic de Carmona (MC), in the state of San Luis Potosí. **a** Yellow cactus prickly pear fruits used when *Opuntia streptacantha* fruits are not available. **b** Stored concentrated juice for the following productions. **c** Woodburning stove with opuntias and agave as fuel. **d** Strainer for seed removal. **e** Metal drum used for colonche boiling. **f** Production site outside the house

In contrast to the Altiplano region, producers from MC invest more time and resources for colonche production, and this process varies among localities. Outside the center of MC, producers boil the fruit juice three times during the preparation process, while the producers in the center of MC only boil the cactus prickly pear fruits one time. Additionally, in this region, prickly pear fruits from different cactus species are included in the mixture (Fig. 3a). Boiling is performed in metallic drums (Fig. 3e), and wooden tools are used to stir the fruit juice while boiling occurs. We recorded that the same wooden tools have been used for colonche production through several generations (80 ± 30 years) (Fig. 3f). Cooking time is not standardized and depends mainly on observations and criteria of the person preparing the product. Boling is performed over a fire from dried agaves and *Opuntias* (Fig. 3c).

The first boiling step allows for the separation of the prickly pear pulp and seeds. The seeds are then removed with a sieve (this is a key step because if seeds are present for the second boiling the product will have a smoky flavor). In a second boiling step, spices are added to give the final product the desired flavor profile. The most common spices used are *Cinnamomum* sp. and *Pimpinella anisum*. After boiling, the juice is filtered to remove the remains of the spices (Fig. 3d). The final boiling step is performed in order to concentrate the sugars. The juice resulting from the final boiling is called “la miel de la tuna” which means the honey from cactus prickly pear fruits. Finally, this concentrated juice can be stored in the fridge in plastic bottles or placed at the clay pot in order to begin the fermentation process (Fig. 3b). It is important to highlight that pulque is commonly used as a starter for colonche fermentation, and this is indeed added to start the fermentation in the concentrated juice.

**Fig. 4:**
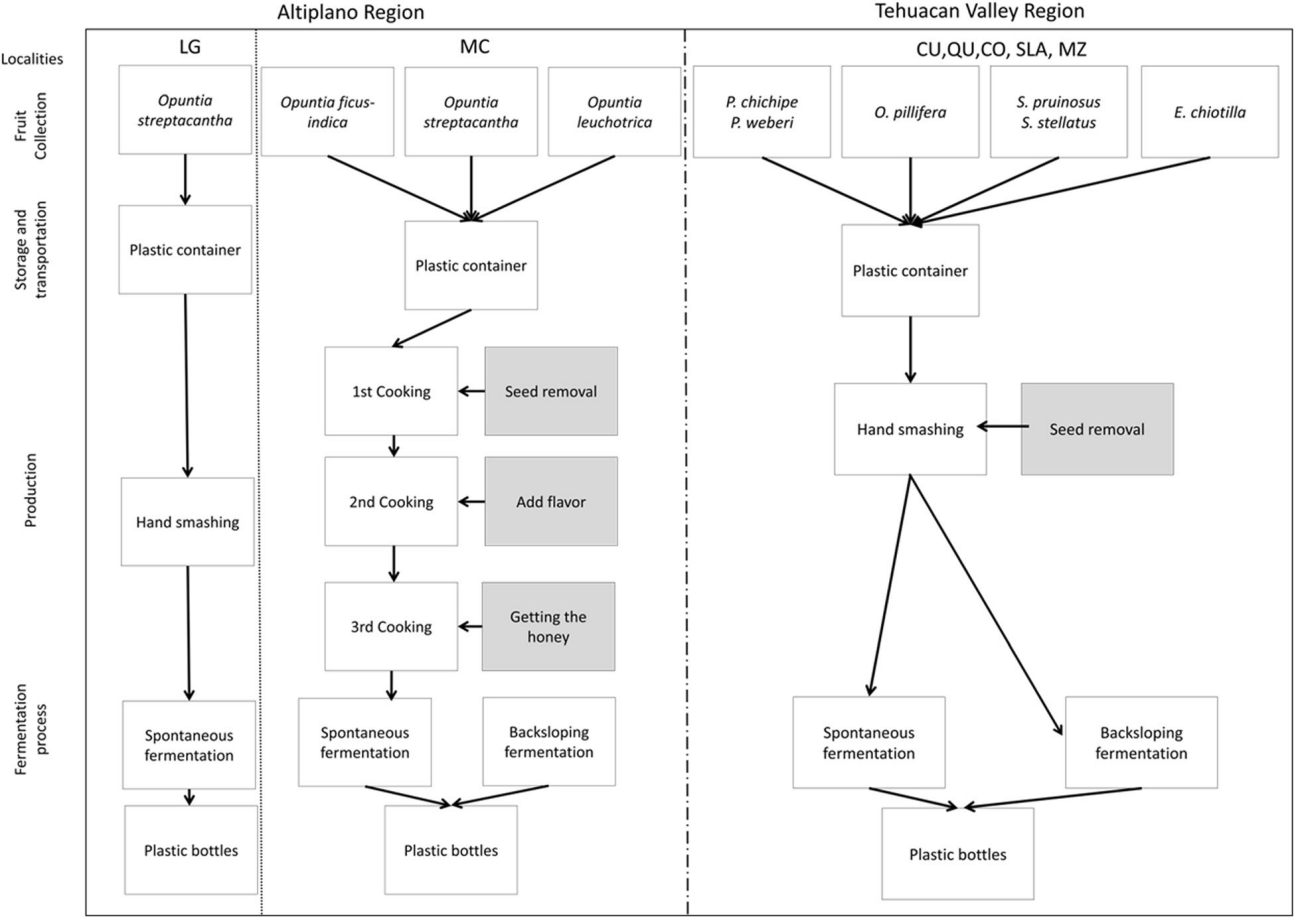
General scheme for colonche production in both regions (Altiplano and Tehuacán Valley), and localities studied; Laguna de Guadalupe (LG), Mexquitic de Carmona (MC), Cuicatlán (CU), Quiotepec (QU), Coaxcatlan (CO), San Luis Atolotitlán (SLA), and Los Reyes Mezontla (MZ)

In the Tehuacán Valley, during the process of separating prickly pear seeds from the pulp, the fruit juice is collected in buckets or clay pots while seeds are rinsed with water and sun-dried. The fruit juice is stored in clay pots and covered, as all cases documented in this study. Then, a small amount of colonche from a previously prepared batch is added as inoculum of fermentation for the new colonche batch. Fermentation is carried out in dark cool rooms and the new colonche batch is ready to drink two or four days later. The fruit production season of columnar cacti is relatively long (3 to 4 months), and colonche may be prepared several times during this season. Seeds of cardón fruits are stored in dry places, packaged, and taken to the regional markets to be sold. People of the Tehuacán-Cuicatlán Valley highly value cardón seeds and typically combine them with chili peppers and green or red tomatoes to make sauces. Also, these seeds can be ground to a butter-like paste used for preparing several dishes, and it may be stored in the fridge to be used throughout the year (Fig. 4).

### Traditional fermentation microorganism’s management

In all the cases studied, the fermentation of colonche occurs within clay plots. In most cases, the clay pot is a part of the household’s cultural heritage and is dated to about 80 years old or older. These pots are not glazed and, when cactus prickly pears fruits are not available, are primarily used for pulque production. Therefore, a layer of microorganisms might always be present in these clay pots. Producers point out that they prefer to produce the fermentation in clay pots rather than plastic or metallic containers because clay pots improve the flavor of the final product. Most of the fermentation occurs at room temperature (about 25 °C to 30 °C).

Fermentation at LG occurs mainly by “spontaneous” fermentation. However, in one case, an experienced colonche producer prepares the first batch of colonche and then shares what is left of this batch to other producers as an inoculated strain known as “zinaiste” or “xinaiste” which guarantees a successful fermentation. The fermentation process lasts for 12 h in LG and 4 h in MC. Fermentation of prickly pear juice in MC starts with the addition of pulque from *Agave salmiana*. Therefore, in this locality, each producer creates their own xinaiste or inoculum. Pulque is added only to the first batch, the relationship is 15 L of concentrated juice and l L of over-pulque, which is called “pulque fuerte” by local people. The xinaiste could be stored in a fridge or added to new batches. The fermentation in MC (4 h) is shorter than in LG (12 h) and could be due to the addition of the microbial community associated with the xinaiste or pulque.

As mentioned above, in the Altiplano localities, water is thought of as a contaminate of colonche, therefore, clay pots and other utensils used for colonche production most be completely dry. In other studies show that water modifies the composition and the dynamics of microorganism’s communities in the fermentation process and can favor the production of acids [39]. Consequently, adding water to the colonche could bring undesirable flavors, modify the color of colonche from purple to brown and acidify the beverage. Careful strategies are carried out in order to store colonche without water. Cleaning occurs at the beginning and end of the season and is performed with only rinsing clay pots with water then sun drying (soap is not used because it has been observed to give a bad taste to the following batches). At the beginning of the season, most of the producers (80%) clean the clay pots with water and then dry them in direct sunlight. Other producers clean the pot with pulque or with a previously stored xinaiste and only 2% of the producers add alcohol to the clay pots and light a match inside the clay pot using fire as a cleaning method.

When colonche availability declines, different strategies are employed to enjoy colonche for an extended period of time. For instance, in LG, colonche can be stored in glass bottles with the addition of alcohol and dried fruits. Producers in MC stored colonche in the fridge at − 4 °C. This method is similarly used in the villages of the Tehuacán Valley where it may be stored in refrigeration and at the end of the season it is commonly kept in the freezer in plastic bags or bottles.

### Selection of colonche desirable flavors

**Fig 5.**
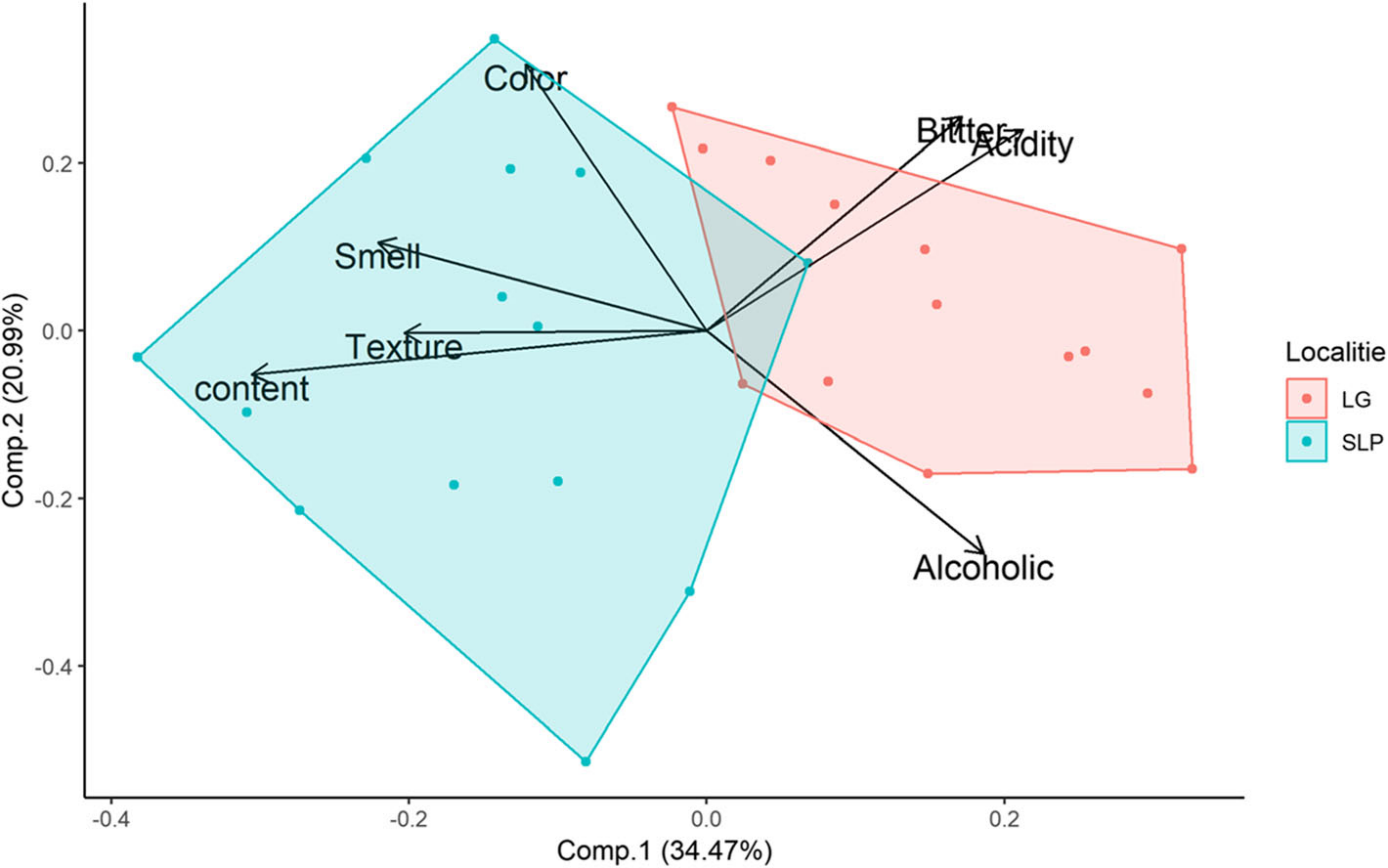
Scatter plot of PCA of sensorial attributes of colonche. Red points represent LG producers and green points MC producers. The axis represents, Principal Component 1 (34.47%) and Principal Component 2 (20.99%), which represent a cumulative variance of 55.46%. Groups of green and red points reflect the differentiation pattern of selection of the sensorial attributes of colonche in the two localities analyzed

As mentioned above, the microbial community of each beverage plays a significant role in shaping flavor attributes (texture, acidity, etc.). These characteristics are selected by the producers and are modified by their practices and processes during the production of the colonche. An evaluation of the sensorial attributes was conducted by ranking from the most important to the less important, respectively. In order to visualize differences between localities, a PCA scatterplot contrasted a priori groups. The results can be visualized in Fig. 5, with the attributes selected by colonche producers from the Altiplano region and the localities. The first axis represents 34.47% of the variance while the second axis represents 20.22% for a total of 54.69% cumulative variance. The attributes of the first principle component (PC1) are sweetness and alcoholic content which apparently generate the highest variance, meanwhile, the attributes of the second principal component (PC2) are color and acidity which seem to explain the rest of variance observed. Clearly, this PCA allows us to separate localities preferences which might be reflected by the differences in their fermentation practices between localities. Particularly, colonche in LG is a sweet beverage with higher alcohol content. On the other hand, MC colonche is favored to have more complex sensorial attributes.

These results give us a glimpse of which microorganisms may be favored under the fermentation of colonche. This work does not directly compare the microbiota in different regions or localities. Further exploration should examine the composition and the structure of the microbiota involved in fermentation in combination with the differences of substrates.

## Discussion

The study of landraces of cultivated plants or rare breeds of livestock has played a central role in ethnobiological studies directed to document management and domestication. In contrast, relatively fewer studies have explicitly specialized in microbial ethnobiology. In this sense, documenting cultural and biological diversity through fermented food recipes, ethnobiologists have a chance to use fermentation to attend the interaction between humans and unseen organisms, the microbial communities. It has been pointed out that fermented products are complex and peculiar expressions of local gastronomies; moreover, they often represent a part of local community identity [40]. In our study, we recorded that colonche production, consumption, and historical identity differs among regions.

Traditional knowledge of fermented foods and beverage production has been recorded in studies around the world [7, 8, 41]. We registered the major role of men in the production of colonche, as the main participants in gathering fruits and producing the beverage. However, women are the main transmitters of knowledge of the practices involved in colonche production. This is thought to be due to the migration of men to other cities of Mexico or the USA. In the locality of PZ, the relatively low number of producers suggests that this beverage is soon to disappear from this locality. The renascence of the colonche tradition in the LG observed in the colonche festival has renewed the interest in consumption and production and has also resulted in a growing number of producers. The renascence of local fermented beverages is made possible by the active role of women, similarly as recorded in other studies [7, 8, 41, 42].

The main goal of the current study was not aimed to describe the health benefits of consuming colonche, but, there is a large body of literature that supports the idea that traditional fermented beverages have positive effects on health [2, 41, 43]. Undoubtedly, nutritional and health benefits of ferments are conferred by microorganisms involved in fermentation [44] and, in this sense, humans have been selecting favorable microorganisms and removing pathogen microorganisms by different strategies since ancient times [45]. Strategies may involve a spectrum of practices from spontaneous fermentation to more specific strategies that control the conditions of fermentation [46–48].

For colonche production, we characterize the strategies such as; selecting specific places for the fermentation, and the usage of temperature to concentrate sugars and flavors; as well as cultural practices like sharing the “seeds” of fermentation and inoculums among communities. It is worth mentioning that the selection and experimenting of new substrates are active processes. For instance, in this study, we documented that people have experimented with preparing colonche with fruits of *Hylocereus undatus* and *Lemairocereus hollianus*, which have been discarded because attributes of the resulting beverages are unsatisfactory.

The ecological impact of colonche production on populations of the species used is yet to be explored. However, it is possible to hypothesize that the impact is low. *O. streptacantha* is cultivated and enhanced in agroforestry systems, while *P. weberi* and *E. chiotilla* are wild species of long-lived arborescent cacti that become dominant in some columnar cacti forests. Previous studies of the population ecology of these species revealed that survival, not fecundity, is the most relevant demographic rate for the maintenance of populations near the equilibrium growth rate lambda [49].

In many cases, we observed in both regions the use of the technique back-slopping to in the fermentation process. Back-slopping is a classical way to improve and optimize the fermentation process by adding microorganisms that are well adapted to the fermentation media to achieve tastier, safer, and healthier products [2, 50–57]. The practice of back-slopping results in the storage of starter cultures for colonche production to be later added to the prickly pear fruit juice. Back-slopping is a common practice for various fermented products, such as sourdough, beer, and other fermented products. This idea is also linked to the interesting phenomenon of the co-evolution of the microorganism community in different fermented goods. It has been registered that when the microbial community is specific to a certain place and type culinary preparations it may result in a long co-evolution between these microorganisms, substrates, and process [8]. Such is the case for some *Penicillium* species in the preparation of cheese [52]. Thus, one can imagine, these preparations may “express” a bio-cultural dimension of a given region or locality [8]. In this sense, we propose that strong and recurrent interactions of ferment good managers and microbial communities might represent possible pathways for the domestication of yeast and bacteria strains. But these processes should be investigated in more depth.

Traditional managers of fermented products do not necessarily name the microorganisms (as they commonly do for animals and plants), but, they do identify and describe with detail the preparation process and the sensorial qualities; taste, smell, and visual aspects [6, 53]. As we have shown, producers not only prefer different sensorial attributes in colonche but also perform active selection throughout the fermentation process in order to achieve such attributes. Additionally, the material of the vessel also influences the final product of colonche. When using a vessel not made from clay, colonche does not have the same quality as is expected by the producers. Experimentation with different types of vessels and substrates tells us that the process of making colonche is still in active exploration. However, the selection of a specific substrate (*O. streptacantha* and *Pachycerus weberi*) by the majority of the producers explains that the selection of sensorial attributes may originate from the potential microorganisms associated with the traditional management of plant material.

Traditional knowledge of fermentation practices is minimal and seemingly undervalued even though this type of knowledge represents a very interesting combination of diverse factors, like; location, practices, local flora, environmental conditions, and microbial taxa. Traditional knowledge also encompasses important cultural components. Mainly, we consider the decreasing transmission of this traditional knowledge from mother-to-son as the main threat to the existence of the practice of making colonche. Particularly, in PZ, we observed that this is rapidly decaying and may be expected to be lost in a couple of generations. In contrast, LG community is actively sharing the practices to prepare and consume colonche. Activities like colonche festival in LG greatly contribute to the preservation of microbial communities used for traditional fermentation, foodways heritage, and human wellbeing. These activities may be significantly supported by scholars and governmental and non-governmental agencies.

## Conclusions

Management practices of fermentation are variable in colonche production, selection of the substrates and the final sensorial attributes of the beverage are different among regions and localities. However, even when microbiota is not seen by producers, the detailed manner in which they describe the preparation and the sensorial qualities such as taste, smell, and alcoholic content of colonche reflect a deep knowledge of the processes influencing fermentation. Our study hopes to build upon socioecological theory for in situ conservation of biodiversity and the cultural knowledge of traditional managers of fermented goods with the hope of encouraging preservation and promoting microbial refugia for these marginalized local foods. Fermentation management is a valuable biocultural heritage that deserves to be documented and protected. It offers the possibility of studying and analyzing the management and domestication processes of unseen organisms and the coevolutionary relationship between the substrates and the microorganisms involved in fermentation.

## Supplementary information

**Additional file 1.** Semi-structured interview for colonche producers

## Data Availability

Please contact the corresponding author for data requests.
