# Supplementary material for: Traditional management of microorganisms in fermented beverages from cactus fruits in Mexico: an ethnobiological approach
Source: J Ethnobiol Ethnomed. 2020 Jan 10;16:1. doi: 10.1186/s13002-019-0351-y (PMC6954596; doi:10.1186/s13002-019-0351-y)
Supplement: Supplementary file 1 — Additional file 1. Semi-structured interview for colonche producers [file 13002_2019_351_MOESM1_ESM.docx]

**Supplemental material**

1. **Semi-structured interview for colonche producers**

__ /__ /20__

**Semi-structured interview for colonche producers.**

**Before you start: remember to say hello, introduce yourself and be polite**

**Explain the objective of the interview and mention the privacy of the information**

Name__________________________ ______________ Age ____________

Locality__________________________________ _______________________

**I. General information**

- **Producer information**

When do you started to produce colonche?_____________________________________________

Why do you produce colonche?_______________________________________________________

Who teach you to produce colonche?__________________________________________________

Do you like to produce colonche (yes / no) ¿why?________________________________________

Did you produce colonche for certain special occasions? _________________________________

How often did you consume colonche? # Times weekly _______________________________

How often did you produce colonche by the week?

Does the production has change since you remember consuming colonche (Yes/No) How?_____________________________________________________________________________

Did you produce something else with cactus prickly pear fruits? ______________________________

Would you like that your beverage continue to be produced by your family?(Yes/no) Why?_____________________________________________________________________________

Do you think that colonche production will continue for the following years? (Yes/no)

Did you know if colonche consumption has health benefits and which ones? __________________________________________________________________________________

Did you prefer colonche instead of another beverages like beer or any other and why?

__________________________________________________________________________________

**II. Production**

- **Production proceess**

From the following scheme which things you recognize and which you don’t apply
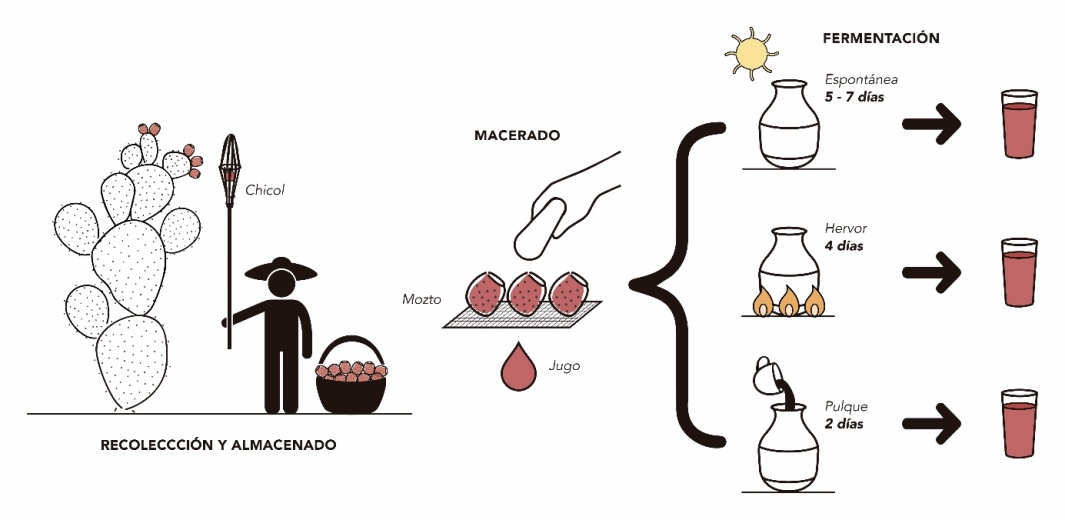


At what time did you go for the cactus prickly pear fruits?

How do you collect the fruits, which instruments do you use?

Did you harvest by your own or did you go with your family?

Did you peel the fruits closer to the collection sites or at your home?

Did you smash the fruits and which utensil you use for smashing?

Where did you perform the fermentation and why do you use it?

Have you try to perform fermentation in another container as plastic or other and what is the main difference?

Is the same recipient year by year and how old is your container?

Do you use this container for any other purposes?

Where the fermentation does takes place inside or outside the house?

- **Plant supplies (substrate)**

Which is the species of cactus prickly pear fruits that you use for and which one is the most valuable for colonche production?

Which characteristics do you choose in the cactus prickly pear fruits: ripen, easy to cut, less prickles, bigger fruits?

How far are those places where you collect the cactus prickly pear fruits?

How much liters of colonche do you have for a bucket (20 lts) of peeled cactus prickly pear fruits?

Did you propagate Opuntias only for colonche production?

When there are no cactus prickly pear fruits available for colonche production where you used to harvest what do you do?

Have you bought cactus prickly pear fruits in order to produce colonche? How much do you buy? And how much does it cost?

1. características _______________________________________________________

- **Cleaning**

Did you use water for colonche production? (Yes /no)

How do you clean your utensils and fermentation container before and after the production?

_____________________________________________________________________________

Do you use soap or another thing in order to clean your container? (Yes/ no) why?

_____________________________________________________________________________

- **Fermentation process**

How long does it take the fermentation process?

Which activities do you perform for colonche production?

Boiling Movements inside the container cover the container addition of other supplies, which ones?____________________________________________________________

Did you use any starter culture for colonche production?_______________________________

Why do you use it and where did you get it?_________________________________________

Do you have any strategy in order to improve your fermentation?________________________

Which things can endanger the fermentation process and how do you avoid it?_____________

How many batches of colonche did you prepare *per* year?

- **Perception of organoleptic attributes.**

To the following attributes rank which ones are more valuable in your beverage?

| Attribute | Value | Description |
| --- | --- | --- |
| Sweetness | 1, 2, 3, 4, 5, 6 ,7, 8 ,9 ,10 | 1 is lees sweet-10 is sweet |
| Texture | 1, 2, 3, 4, 5, 6 ,7, 8 ,9 ,10 | 1 is lees complex-10 is more complex |
| Odor | 1, 2, 3, 4, 5, 6 ,7, 8 ,9 ,10 | 1 is less pleacent- 10 is more pleacent |
| Alcoholic content | 1, 2, 3, 4, 5, 6 ,7, 8 ,9 ,10 | 1 is less alcoholic-10 is more alcoholic content |
| Acidity | 1, 2, 3, 4, 5, 6 ,7, 8 ,9 ,10 | 1 is less acid – 10 is more acid |
| Color | 1, 2, 3, 4, 5, 6 ,7, 8 ,9 ,10 | 1 is not particularly important- 10 is really red |
| Other | 1, 2, 3, 4, 5, 6 ,7, 8 ,9 ,10 |  |

- **Economical motivations**

Does it represents incomes for your family?______________________________________________

Where did you sell your product?______________________________________________________

How much does a liter cost?___________________________________________________________

**Any other commentaries at the end of the interview ________________________________________________________________________________________________________________________________________________________________________________________________________________________________________________________________________________________________________________________________________**

**Remember to thank and say goodbye.**
